# Retraction: Seven-gene signature based on glycolysis is closely related to the prognosis and tumor immune infiltration of patients with gastric cancer

**DOI:** 10.3389/fonc.2024.1490596

**Published:** 2024-09-17

**Authors:** Frontiers Editorial Office

The journal retracts the 2020 article cited above.

Following publication, Frontiers was alerted to the presence of non-verifiable cell line identifiers in this publication. Upon further examination, it was determined that core findings of this study were obtained using non-verifiable cell lines and/or cell lines known to be cross-contaminated with HeLa. This contamination compromises the data and conclusions of the article and the article has been retracted.

This retraction was approved by the Chief Executive Editor of Frontiers. The authors received a communication regarding the retraction and do not agree to this retraction. This communication has been recorded by the publisher.

